# Liver Transcriptomic Reveals Novel Pathways of Empagliflozin Associated With Type 2 Diabetic Rats

**DOI:** 10.3389/fendo.2020.00111

**Published:** 2020-03-17

**Authors:** Qiuyue Lv, Liang Le, Jiamei Xiang, Baoping Jiang, Sibao Chen, Peigen Xiao

**Affiliations:** ^1^Institute of Medicinal Plant Development, Chinese Academy of Medical Sciences, Peking Union Medical College, Beijing, China; ^2^Institute of Chinese Materia Medica, China Academy of Chinese Medical Sciences, Beijing, China

**Keywords:** empagliflozin, liver, glucose metabolism, type 2 diabetes, RNA-seq analysis

## Abstract

The hypoglycaemic target of empagliflozin (EMP), as a novel inhibitor of sodium-glucose cotransporter (SGLT2), is clear. However, recent studies have shown that EMP also has an important role in lipid metabolism and cardiovascular diseases. The liver plays an important role in the development of type 2 diabetes (T2D), although whether EMP affects liver glucose metabolism is currently not reported. This study was designed to evaluate the effect of EMP on hepatic glucose metabolism in T2D and the underlying mechanism. A model of T2D was established by a high-fat and glucose diet (HFD) combined with streptozotocin (30 mg/kg) in male Wistar rats. Serum samples were collected to measure biochemical indicators, and liver samples were extracted for RNA-seq assay. Quantitative real-time PCR (qPCR) was used to further verify the gene expression levels detected by the RNA-seq assay. The EMP group showed significantly decreased blood glucose, triglyceride, cholesterol, non-esterified fatty acid and low-density lipoprotein cholesterol levels, and increased high-density lipoprotein cholesterol levels in serum compared with the type 2 diabetes model (MOD) group. Furthermore, EMP decreased the levels of inflammatory factors IL-1β, IL-6, and IL-8 in the serum compared to the MOD. Liver transcriptome analysis showed EMP affects a large number of upregulated and downregulated genes. Some of these genes are novel and involve in the metal ion binding pathway and the negative regulation of transcription from the RNA polymerase II promoter pathway, which are also closely related to glucolipid metabolism and insulin signaling. Our study provides new knowledge about the mechanism through which SGLT inhibitor can offer beneficial effects in T2D and especially in the hepatic metabolism. These genes found in this study also laid a solid foundation for further research on the new roles and mechanisms of EMP.

## Introduction

Type 2 diabetes (T2D) poses an enormous burden on patients and the health care system and is one of the leading causes of death worldwide. According to a recent survey, the global prevalence of diabetes was 425 million people in 2017 and is expected to increase to 629 million by 2045 (1). T2D is a metabolic disorder that is characterized by hyperglycemia induced by impaired glucose tolerance, low-grade inflammation and insulin resistance (2), and accounts for ~90% of the total prevalence of diabetes. The liver is the major involved in glucose and lipid metabolism, playing an important role in the development of T2D (3). When the liver is fatty, the ability of insulin to inhibit glucose production is impaired (4, 5). Therefore, studying the role of drugs in the prevention and treatment of T2D is important from the perspective of hepatic glycolipid metabolism.

Empagliflozin (EMP) is a novel inhibitor of the sodium-glucose cotransporter (SGLT2). EMP, which is highly selective, lowers blood glucose levels by reducing the reabsorption of glucose in the kidneys and increasing the excretion of glucose in the urine and has been approved for T2D treatment in the EU and US (6). When large amounts of glucose are forced into urinary excretion processes, whole-body metabolism undergoes adaptive changes involving glucose fluxes, hormonal responses, and fuel selection (7). Current research has shown that EMP suppressed weight gain by enhancing fat utilization and browning and attenuated obesity-induced inflammation and insulin resistance by polarizing M2 macrophages in white adipose tissue (WAT) and liver (8). Furthermore, EMP alleviated diabetic cardiac microvascular injury by inhibiting mitochondrial fission via the activation of AMPK signaling pathways (9).

Although the target of EMP as a hypoglycaemic agent is clear, recent studies have shown that it plays an important role in lipid metabolism and cardiovascular diseases. As the main target organ of glycolipid metabolism, the liver has an important role in T2D. However, research on whether EMP affects glucose metabolism in the liver is lacking. In this study, metformin (MET) was used as a positive control drug, and we aimed to investigate the effect of EMP on hepatic glucose metabolism in T2D rats. RNA-seq analysis was used to identify differentially expressed genes (DEGs) in liver transcripts from T2D rats to investigate the mechanism by which EMP affect hepatic glucose metabolism.

## Methods

### Animal Procedure and Drug Treatment

Forty male Wistar rats weighing 200 ± 20 g were purchased from Speyford (Beijing) Biotechnology Co., Ltd. All rats were maintained under a 12 h light/dark cycle at room temperature (22 ± 2°C) with free access to food and water. The animal experiments were performed in accordance with the Guidelines and Policies for Animal Surgery under the approval of the Chinese Academy of Medical Sciences and Peking Union Medical College, Beijing, China (approval No: SLXD-20170302526) and were approved by the Institutional Animal Use and Care Committee (IACUC)(10).

After 7 days of acclimatization, rats were randomly divided into four groups with 10 rats per group as follows: control group, type 2 diabetes model (MOD) group, MET group, and EMP group. The latter three groups were fed a high-fat and glucose diet (HFD; 45% fat, 20% protein, and 35% carbohydrate) for 4 weeks and then given a single intraperitoneal (i.p.) injection of streptozotocin (30 mg/kg, freshly prepared in citrate buffer, pH 4.5) after overnight fasting. The control group was fed standard rat chow (12% fat, 28% protein, 60% carbohydrate) and injected with the same dose of citrate buffer. HFD feeding continued for another 4 weeks, and then blood samples had collected from the tail vein for blood glucose level analysis. Thereafter, rats in the MET and EMP groups were intragastric administration with MET (200 mg/kg/d) and EMP (10 mg/kg/d), respectively, for 4 weeks. At the end of treatment, all rats were fasted for 8 h and terminally anesthetized with 1% sodium pentobarbital (40 mg/kg); blood samples were collected from the abdominal aorta, and plasma was separated for biochemical assessment. In addition, the livers were quickly excised and stored at −80°C for subsequent analysis.

### GTTs and ITTs

A glucose tolerance test (GTT) was conducted after an overnight fast, and the rats were injected i.p. with 2 g/kg glucose. An insulin tolerance test (ITT) was performed after a 4 h fast, and rats were injected i.p. with 0.75 U/kg recombinant human insulin. Blood samples were collected from the tail vein, and blood glucose was measured by a hand-held glucometer (Roche, China) at 0, 30, 60, 90, and 120 min.

### Biochemical Analyses

Serum samples were collected to measure fasting blood glucose (GLU), non-esterified fatty acid (NEFA), triglycerides (TG), cholesterol (CHO), high-density lipoprotein cholesterol (HDL-c), low-density lipoprotein cholesterol (LDL-c), insulin (INS), tumor necrosis factor (TNF-α), and interleukin (IL-1β, IL-6 and IL-8) levels. Whole blood samples were collected to measure hemoglobin (HbA1c) level. GLU, TG, CHO, HDL-c, and LDL-c were measured using their respective diagnostic kits (Biosino Bio-Technology and Science Inc., China). NEFAs were estimated using a NEFA test kit (Beijing Strong Biotechnologies, Inc., China). INS, TNF-α, IL-1β, IL-6, and IL-8 were measured using an ELISA kit (Biosource, USA) according to the manufacturer's protocol. HbA1c was measured using an HbA1c test kit (Beijing Wantai Dro Co., Ltd., China).

Homeostasis model assessment-insulin resistance (HOMA-IR) was used to assess insulin resistance from basal glucose and insulin. The HOMA-IR index was calculated using the following equations: HOMA-IR = [fasting glucose (mmol/L) × fasting insulin (μU/mL)]/22.5(11).

### Haematoxylin-Eosin (H.E.) Staining

The pathologic specimens were fixed for at least 24 h with 4% buffered neutral formalin solution and then embedded in paraffin wax for histopathological evaluation. The liver sections were stained with H.E. and observed under a light microscope.

### Total RNA Preparation

Total RNA was extracted from each liver sample using an RNAiso plus kit, according to the manufacturer's instructions. RNA degradation and contamination were monitored on 1% agarose gels. The purity of each RNA sample was determined using a NanoPhotometer® spectrophotometer (IMPLEN, CA, USA), the concentration of each RNA sample was determined using a Qubit® RNA Assay Kit with a Qubit 2.0 fluorometer (Life Technologies, CA, USA), and the integrity was determined using the RNA Nano 6000 Assay Kit of the Bioanalyzer 2100 system (Agilent Technologies, CA, USA). A portion of the total RNA was used for RNA sequencing, and the rest was reverse transcribed into first-strand cDNA using MMLV reverse transcriptase (Cat. # M1705, Promega, Wisconsin, USA), oligo (dT15) primers (Cat. # C1101, Promega, Wisconsin, USA) and a dNTP mixture (Cat. # U1515, Promega, Wisconsin, USA).

### RNA-Seq and Data Analysis

Sequencing libraries were generated using NEBNext® Ultra™ RNA Library Prep kit for Illumia® (NEB, USA) following the manufacturer's recommendations, and index codes were added to attribute sequences to each sample. All samples were sequenced (paired ends, 100 bp) on the Illumina HiSeq 4000 platform, and 125-bp/150-bp paired-end reads were generated. In this study, we report RNA-seq analysis results for the control, MOD, MET, and EMP groups, with each group containing five samples.

TopHat v2.0.12 was used to align transcript sequences obtained from RNA sequencing to the UCSC reference genome rn6. Cufflinks was then used to estimate the transcript levels (expected number of fragments per kilobase of transcript sequence per millions base pairs sequenced, FPKM) of the RefSeq genes. DEGs were identified using Cuffdiff with the default parameters at *p* < 0.05 for the four groups.

### Gene Ontology (GO) and Kyoto Encyclopedia of Genes and Genomes (KEGG) Enrichment Analyses of DEGs

The GO enrichment analysis of DEGs was implemented by the GOseq R package, in which gene length bias was corrected. DEGs were considered significantly enriched in GO categories that had a corrected P value less than 0.05. We used KOBAS software to test the enrichment of DEGs in KEGG pathways.

### Quantitative Reverse Transcription Real-Time PCR (qPCR)

cDNA was prepared using a TaKaRa® PrimeScriptTM RT Reagent Kit (Perfect Real Time), and PCR was performed using a 2 μl sample from each reverse transcription reaction in a final volume of 25 μl [TaKaRa® TB GreenTM Ex TaqTM II (Til RNaseH Plus)]. Reactions were performed in a CFX96TM Real-Time PCR Detection System (Bio-Rad) at 95°C for 30 s, followed by 40 cycles of 95°C for 5 s and 60°C for 10 s. The primers used for real-time PCR are listed in Table 1.

**Table 1:** Primers used for real -time PCR amplification.

**Gene**	**Forward primer**	**Reverse primer**
*Irs1*	CAT GAG CCC CAA GAG TGT ATC	CCA ATG TCA GGA GAG CAA CTA C
*Akt2*	CCG AGT CCT ACA GAA TAC CAG	ACT CCA TCA CAA AGC ACA GG
*Hmbs*	ACC AAG GAG CTA GAA AAC GC	CAG CAT CAC AAG GGT TTT CC
*Il6r*	TGA AGA CTA TGA CAA CCA CGA G	CAC AGA GAA GCA ATC CAA ACG
*Jak1*	TCT GAT GTC TGG TCT TTT GGA G	AGC CGT GTT ACT GTC ATC TG
*Rheb*	GGA AAG GCT TTG GCA GAA TC	ACG AAG ACT TCC CTT GTG AAG
*Rptor*	TTC CAC CCC TTT ACA CCA TG	CCA TTC AGG TAC TCC ATA GCA G
*Arl1*	TTGACAGTTGTGACCGAGAC	ATTTCTGAGGGCGTCATGG
*Atp2a2*	TTTGGGCAGGATGAGGATG	TTGTGGGAAGGTTCAACTCG
*Cpped1*	CTACTTCAACCTCACCAAGACC	ACCCGATGGCAGATGAAAC
*Enpp2*	TTAAGAGGGCAGAATGGGATG	TTACACAGGTTGTCACACCG
*Klf10*	GAAGTCACATCTGTAGCCACC	TCCTTTCACAGCCTTTCCAG
*Ndufa4*	TCG TGT TTA TTG GAG CAG GG	ACC CAG TTT GTT CCA AGG C
*Ndufa2*	CAT TCA CTT ATG CCA GCG TTC CCG	GGA TTA GAA TGG GCA GGT CG
*Sox17*	CAC AGC ATA GTG TCT ACA GGC	CGA GAG CGT GCA GGA AG
*Pax5*	CCA TCC CAA CTA CAA GTA	AAT ACC TTC ATC CCT CTT GCG
*Nsf*	AGGAAATGGAGATGCTCAACG	TCTTCCCTTTGACTTGCTGAG
*Vav2*	AAGTGAAACGGGACAAGGAG	TTGGTGTGGTTGACTATGGAC
*Sirt2*	GACGAGCTGACCCTTGAAG	TCTCCAAGTTTGCATAGAGGC

### Statistics and Network Analysis

Data are presented as means ± SEM. Statistical analysis was conducted with SPSS 25.0 software. One-way ANOVA followed by Fisher's PLSD *post-hoc* test was performed when data involved in all four groups (control, MOD, MET and EMP). A two-sample unpaired Student's *t*-test was used for two-group comparisons in gene expression results detected by qPCR. Statistical significance was set at *p* < 0.05. The method used for network analysis was described in our previous work (10).

## Results

### Effect of EMP on Body Weight, Food, and Water Intake

After HFD treatment for 4 weeks, the weight of rats in the MOD, MET, and EMP groups increased but was not significantly different from that of rats in the control group. After streptozotocin injection, the weight of the control group rats increased naturally, while the weight of rats in the MOD, MET, and EMP groups significantly decreased. MET and EMP treatment had no significant effect on body weight compared to MOD group (Supplementary Figure 1A). Compared with the MOD group rats, Wistar rats in the MET group had no significant effect on food and water intake (Supplementary Figures 1B,C). Compared with the MOD group rats, rats treated with EMP exhibited no significant change in food and water intake (Supplementary Figures 1B,C).

### Effect of EMP on Parameters of Glucose, Lipid Metabolism, and Inflammation

As shown in Table 2, rats in the MOD group had a significant increase (*p* < 0.05) in blood glucose, HbA1c, TG, CHO, TNF-α, IL-1β, IL-6, and IL-8 levels compared to the control rats. Furthermore, the serum levels of LDL-c and NEFA were higher in the MOD group than in the control group. Compared with the MOD group, oral administration of MET significantly decreased (*p* < 0.05) the levels of blood glucose, HbA1c, TG, CHO, NEFA, IL-1β, IL-6, and IL-8, and partially reduced the level of TNF-α and increased the level of HDL-c. Compared with the MOD group, oral administration of EMP significantly decreased (*p* < 0.05) the levels of blood glucose, LDL-c, TG, CHO, and NEFA, partially decreased the levels of HbA1c, TNF-α, IL-1β, IL-6, and IL-8, and increase the level of HDL-c.

**Table 2:** Serum parameters in control group of rats and treated rats.

	***In vivo*** **treatment group**			
	**CON**	**MOD**	**MET**	**EMP**
Glu[mmol/L]	5.30 ± 1.40	18.90 ± 5.24^****^	10.11 ± 3.15##	9.64 ± 2.89##
HbA1c [%]	6.01 ± 0.65	7.31 ± 0.60^**^	6.49 ± 0.33#	7.13 ± 0.58
HDL-c [mmol/L]	0.66 ± 0.25	0.61 ± 0.22	0.56 ± 0.17	0.73 ± 0.32
LDL-c [mmol/L]	0.21 ± 0.07	0.56 ± 0.49	0.12 ± 0.03#	0.14 ± 0.03#
TG[mmol/L]	0.49 ± 0.31	4.04 ± 3.88^*^	0.58 ± 0.44#	0.60 ± 0.28#
CHO[mmol/L]	1.05 ± 0.47	2.44 ± 1.68^*^	0.82 ± 0.25 #	1.04 ± 0.42#
NEFA[mmol/L]	0.73 ± 0.24	1.09 ± 0.59	0.52 ± 0.17#	0.55 ± 0.15#
TNF-α(pg/ml)	46.66 ± 4.31	64.51 ± 19.07^*^	45.96 ± 17.58	60.17 ± 16.59
IL-1β(pg/ml)	15.16 ± 1.39	21.94 ± 7.58^*^	15.79 ± 3.29#	16.38 ± 2.27
IL-6(pg/ml)	116.69 ± 17.24	161.86 ± 13.38^****^	121.42 ± 24.76##	148.04 ± 17.94
IL-8(pg/ml)	41.05 ± 3.99	52.80 ± 7.04^**^	32.99 ± 9.04##	49.05 ± 9.65

* *p < 0.05*,

** *p < 0.01*,

**** *p < 0.0001 vs. Control; #p < 0.05, ##p < 0.01 vs.MOD. Data are presented as means ± SEM*.

**Figure 1:**
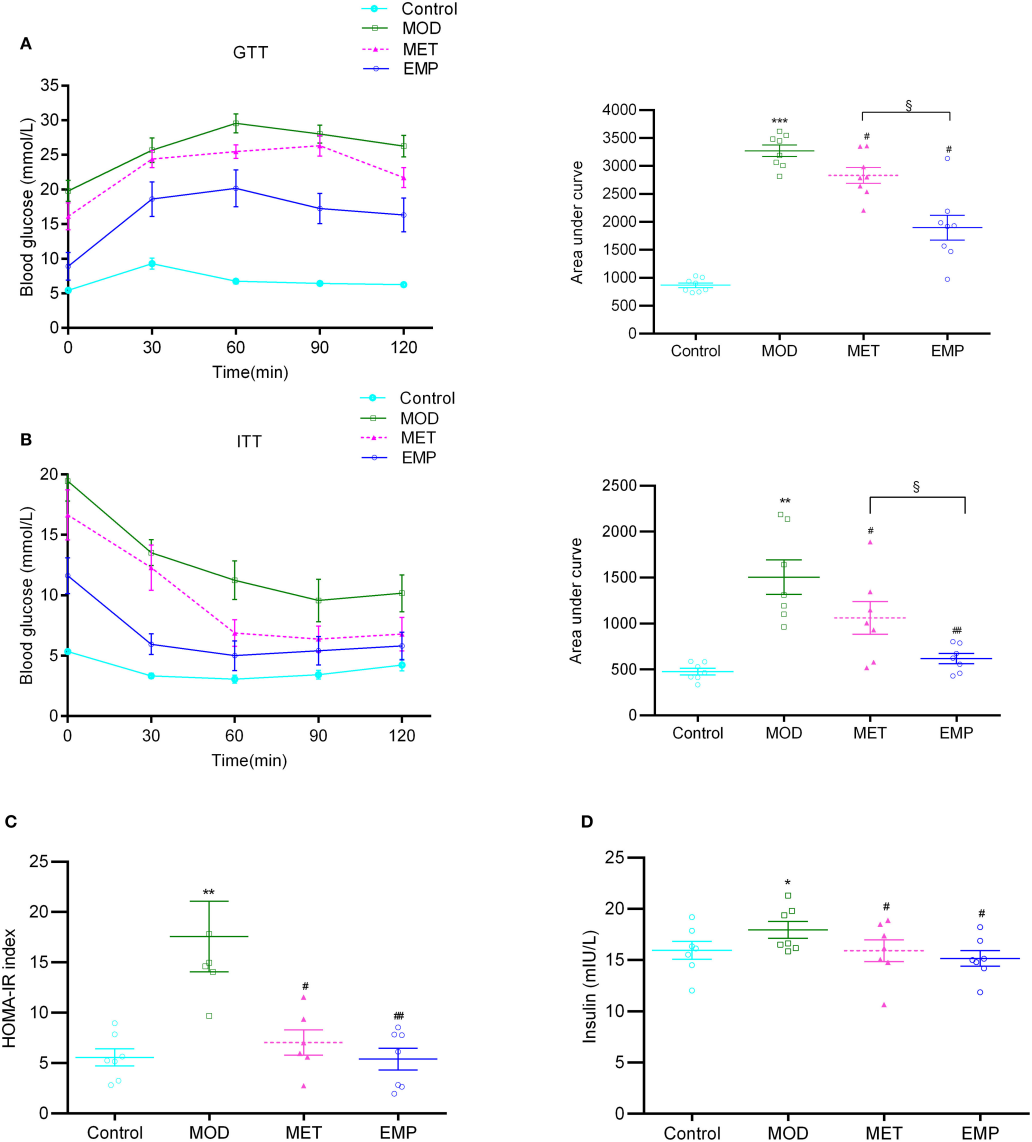
The impact of EMP and MET on glucose tolerance and insulin resistance. **(A)** The oral glucose tolerance test (OGTT) changes in blood glucose levels over time after oral administration of 2 g/kg glucose. **(B)** The blood glucose area under the curve (AUC) represents the insulin tolerance test (ITT) changes in blood glucose over time after intraperitoneal injection of 0.75 U/kg recombinant human insulin. **(C)** Homeostasis model assessment-estimated insulin resistance (HOMA-IR) index. **(D)** A representation of the fasting insulin levels. *p < 0.05, **p < 0.01, ***p < 0.001 vs. Control; #*p* < 0.05, ##*p* < 0.01 vs. MOD; §*p* < 0.05 vs. MET.

Compared with the MOD group, the EMP group showed a significant decrease (*p* < 0.05) in the AUC-GTT and AUC-ITT, indicating that EMP could ameliorate glucose intolerance and increase insulin sensitivity (Figures 1A,B). Furthermore, EMP significantly reduced (*p* < 0.05) the HOMA-IR index and decreased the serum insulin level (Figures 1C,D), indicating that EMP could suppress hyperinsulinemia and improved insulin resistance. MET followed a trend similar to EMP; however, the ability of EMP to improve glucose tolerance and insulin sensitivity was significantly better (*p* < 0.05) than that of MET (Figures 1A,B).

### Liver Pathological Changes Observed by H.E. Staining

**Figure 2:**
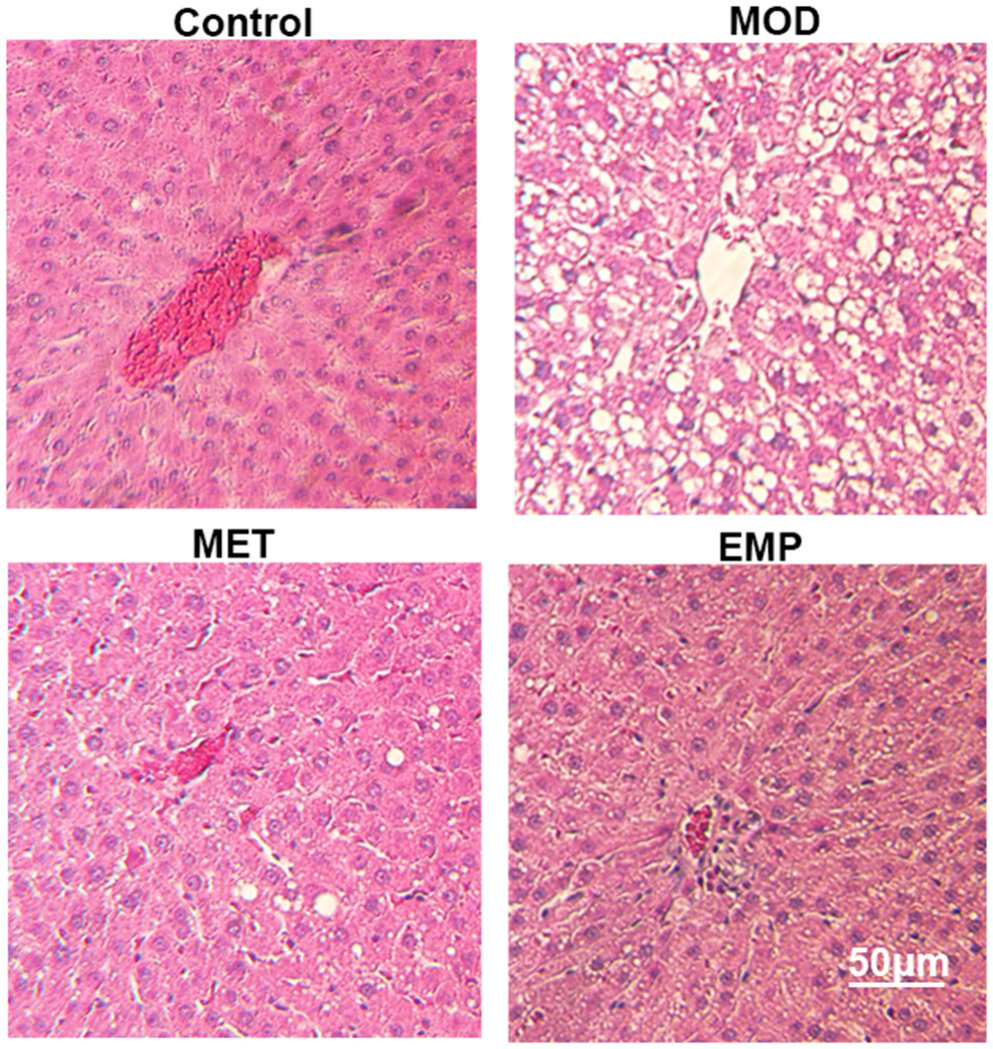
The impact of MET and EMP on liver pathological changes. The liver sections were stained with H.E. and one representative photo of the three rats in every group is shown.

In the control group, the liver showed a completely normal structure with a rounded nucleus at the center of each cell with distinguishable edges and a clear outline (Figure 2). However, in the MOD group, the structure of the hepatic lobules was unclear, most of the cells were swollen and varied in size, and the edges were blurred. The cytoplasm was filled with different-sized fat vacuoles, pushing the nucleus to the periphery, and displaying significant fat changes (Figure 2). Oral administration of MET and EMP significantly reduced the fat vacuoles and improved hepatic steatosis (Figure 2).

### MOD Induces Sustained Transcriptional Changes That Are Partly Reversed by EMP

**Figure 3:**
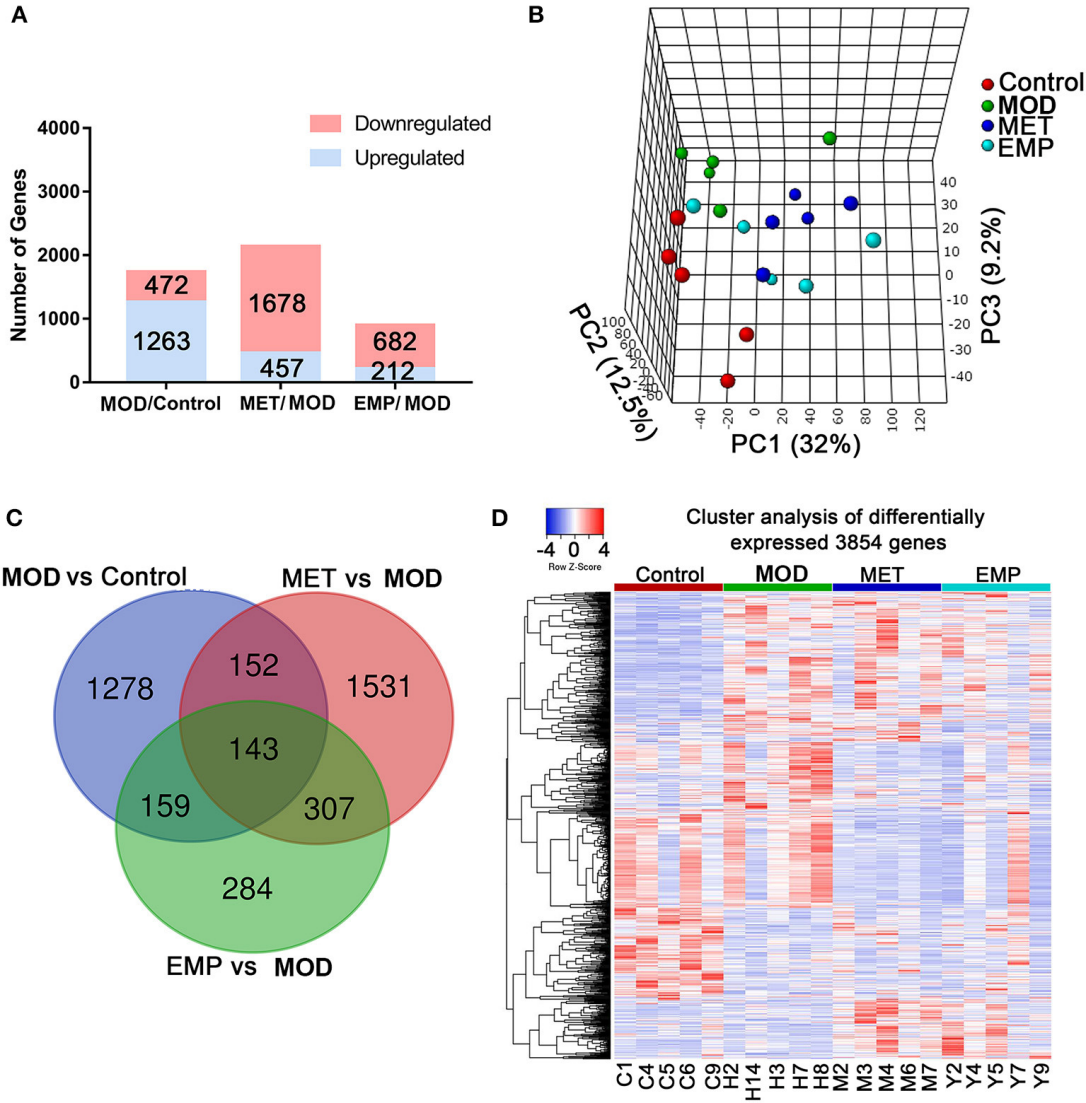
MET and EMP alter liver transcription. Rat livers were collected and then subjected to RNA-seq gene expression analysis. **(A)** The number of upregulated (blue) and downregulated (red) genes from the MOD/control, MET/MOD, and EMP/MOD categories in the rat liver genome. **(B)** 3D view of PLS-DA analysis of the control, MOD, MET, and EMP groups. **(C)** The Venn diagram shows an overlap among the MOD/control, MET/MOD, and EMP/MOD genes. **(D)** Heat maps of the most differentially expressed genes from control, MOD, MET, and EMP comparisons. Each column represents an individual animal.

To understand the molecular mechanisms by which EMP alleviates diabetes, we performed transcriptome profiling of the liver in four groups. Rigorous bioinformatic and statistical approaches were used to analyze the RNA-seq data from the control, MOD, MET, and EMP group samples. DEGs were identified using three comparisons: control/MOD, MET/MOD, and EMP/MOD (Figure 3). This experimental design allowed us to identify DEGs whose expression levels were reversed or resistant to MET and EMP. There were 1735 DEGs in the MOD/control comparison (total gene counts: DEG_up_ = 1,263; DEG_down_ = 472; Figure 3A). Furthermore, in the MET/MOD comparison, there were 2135 DEGs (total gene counts: DEG_up_ = 457; DEG_down_ = 1,678; Figure 3A). In addition, in the EMP/MOD comparison, there were only 894 DEGs (total gene counts: DEG_up_ = 212; DEG_down_ = 682; Figure 3A). DEGs were analyzed by partial least squares-discriminant analysis (PLS-DA), as the PLS-DA map can visually show the classification effect between different groups, and the greater the degree of separation is between the sample groups, the more significant the classification effect. According to the PLS-DA three-dimensional score map, the MOD group and the control group showed a spatial separation trend, suggesting a significant difference in gene expression (Figure 3B). However, the MET and EMP groups were spatially distinct from the MOD group, and approached the control group, indicating that MET and EMP treatment significantly changed the gene expression (Figure 3B).

We identified 1,732 DEGs in the comparison of MOD vs. control groups, 2,133 DEGs in the MET-treated vs. MOD groups, and 893 DEGs in the EMP-treated vs. MOD groups (Figure 3C). Of the genes significantly affected by the MOD, 1,437 genes remained dysregulated after MET treatment and 1,431 genes remained dysregulated after EMP treatment (Figure 3C). A total of 295 DEGs emerged in MET vs. MOD and MOD vs. control, and 302 DEGs emerged in EMP vs. MOD and MOD vs. control (Figure 3C). Genes with similar expression patterns often have similar functions or are involved in the same metabolic pathways (12). To identify clusters with functional enrichment, hierarchical clustering was performed based on gene expression patterns (Figure 3D). The gene expression profile showed clear differences among the control, MOD, MET, and EMP groups.

### The Genes Most Downregulated by MET and EMP Compared With MOD

**Figure 4:**
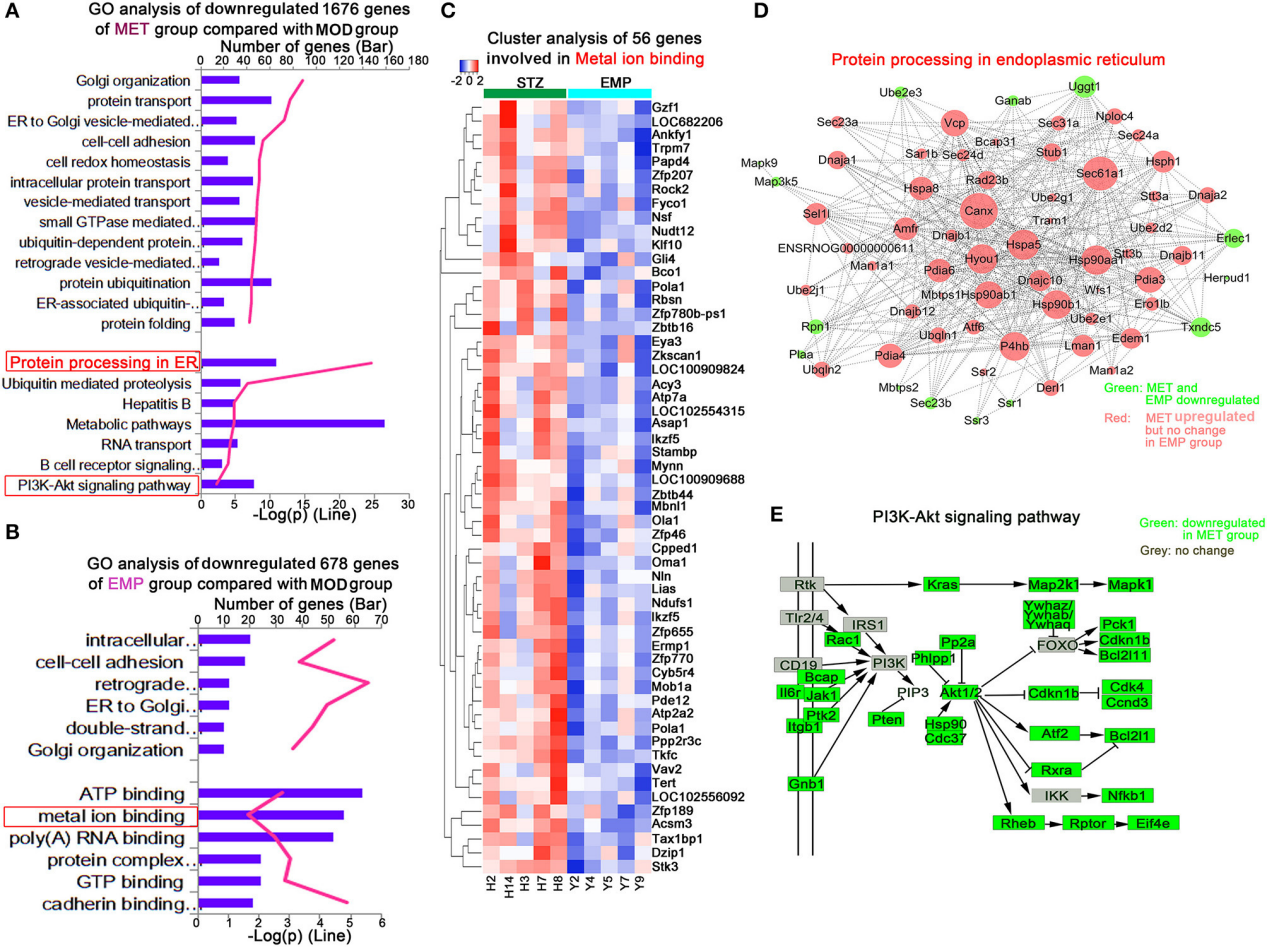
MET and EMP downregulate genes. **(A)** Functional enrichment of MET/MOD-specific DEGs with the highest FDR values. **(B)** Functional enrichment of EMP/MOD-specific DEGs with the highest FDR values. **(C)** Heat maps of most DEGs involved in metal ion binding. **(D)** Protein processing in the endoplasmic reticulum common gene network indicates DEGs downregulated by MET and EMP (green) and DEGs downregulated by MET but not by EMP (red). **(E)** The PI3K-Akt signaling pathway. Each column represents one individual animal.

We identified 1,676 genes whose expression levels were significantly downregulated in the MET group compared with the MOD group and 678 genes whose expression levels were significantly downregulated in the EMP group compared with the MOD group (Figures 4A,B). Functional enrichment was performed using Metacore^TM^, and the results showed that these DEGs affected by MET were enriched in GO categories mainly associated with protein processing in the endoplasmic reticulum and the PI3K-Akt signaling pathway (Figure 4A); however, DEGs affected by EMP were enriched in GO categories mainly associated with metal ion binding (Figure 4B). The 56 downregulated genes affected by EMP were related to several biological processes, and some of these genes, including N-ethylmaleimide-sensitive factor (*Nsf*), Kruppel-like factor 10 (*Klf10*), calcineurin-like phosphoesterase domain containing 1 (*Cpped1*), nucleotide pyrophosphatase/phosphodiesterase 2 (*Enpp2*), ATPase sarcoplasmic/endoplasmic reticulum Ca2+ transporting 2 (*Atp2a2*) and vav guanine nucleotide exchange factor 2 (*Vav2*), are involved in glucose metabolism (Figure 4C). Network interactions showed the DEGs involved in protein processing in the endoplasmic reticulum. Green indicates genes downregulated by MET and EMP, while red indicates genes upregulated by MET (Figure 4D). Furthermore, MET-downregulated genes are mainly involved in the PI3K-Akt signaling pathway, such as interleukin 6 receptor (*Il6r*), Janus kinase 1 (*Jak1*) and AKT serine/threonine kinase 1 (*Akt1*) (Figure 4E). The above results indicate that MET mainly affects protein processing in the endoplasmic reticulum and the PI3K-Akt signaling pathway, while EMP has little effect on protein processing in the endoplasmic reticulum and the genes downregulated by EMP are mainly involved in metal ion binding. Thus, EMP and MET have very different activity pathways.

### The Genes Most Upregulated by MET and EMP Compared With MOD

**Figure 5:**
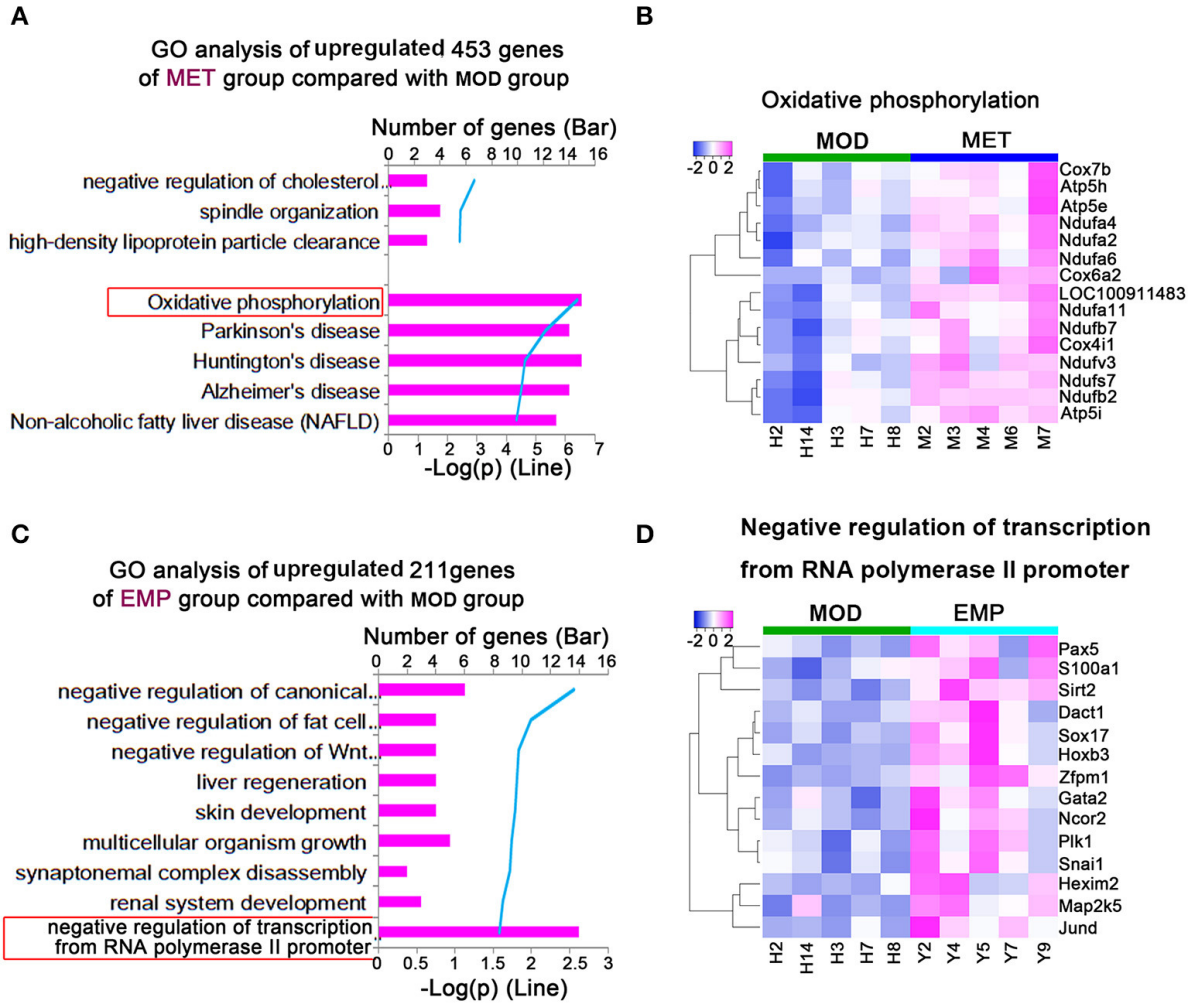
MET and EMP upregulate genes. **(A)** Functional enrichment of MET/MOD-specific DEGs with the highest FDR values. **(B)** Heat maps of most DEGs involved in oxidative phosphorylation. **(C)** Functional enrichment of EMP/MOD-specific DEGs with the highest FDR values. **(D)** Heat maps of the most DEGs involved in negative regulation of transcription from the RNA polymerase II promoter. Each column represents one individual animal.

We identified 453 genes whose expression levels were significantly upregulated in the MET group compared with the MOD group. To explore differences in biological processes between the MET and MOD groups, DAVID6.8 was used to perform gene function enrichment analysis based on GO and KEGG annotations for the significantly DEGs in both groups (Figure 5). The main biological processes identified for the MET group were related to oxidative phosphorylation (Figure 5A). Our results showed that 15 genes in the MET/MOD comparison were involved in oxidative phosphorylation, including multiple isoforms of cytochrome c oxidase, such as *Cox7b, Cox4i1*, and *Cox6a2*, subunits of mitochondrial ATP synthase, such as *Atp5h, Atp5e*, and *Atp5i*, and many isoforms of mitochondrial complex I, such as *Ndufa4, Ndufa2, Ndufa6*, and *Ndufb2* (Figure 5B).

We identified 211 genes whose expression levels were significantly upregulated in the EMP group compared with the MOD group. The main biological processes identified for the EMP group were related to the negative regulation of transcription from the RNA polymerase II promoter (Figure 5C). Our results showed that 14 genes in the EMP/MOD comparison were involved in the negative regulation of transcription from the RNA polymerase II promoter, such as paired-box 5 (*Pax5*), sirtuin 2 (*Sirt2*), and SRY box 17 (*Sox17*) (Figure 5D).

### Validation of Differential Gene Expression Profiles by Quantitative Real-Time PCR (qPCR)

**Figure 6:**
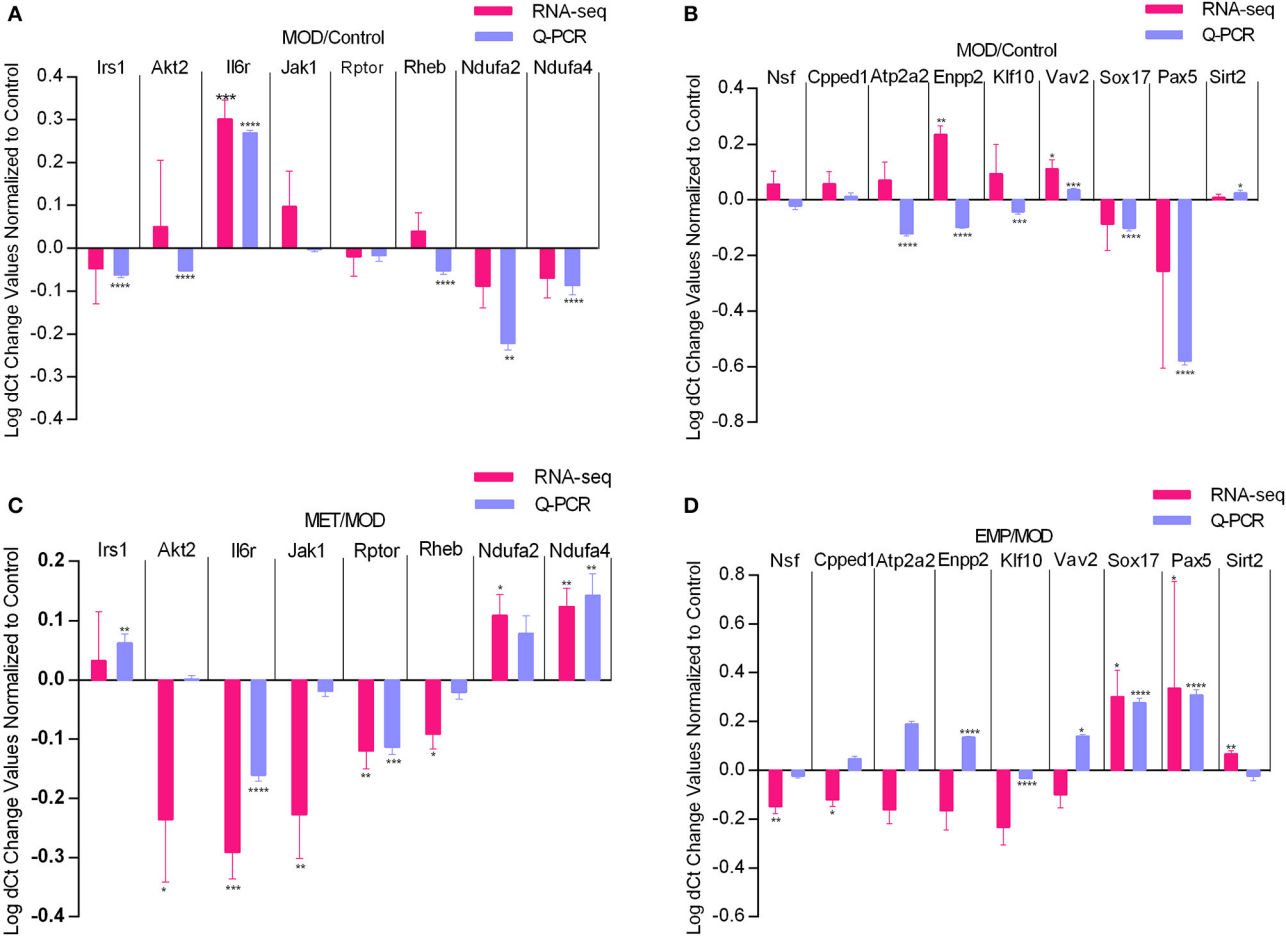
Quantitative real-time PCR validation of RNA-seq analysis of DEGs affected by MET and EMP treatment. Graphs represent logarithmic mean fold changes for MOD values normalized to those of the control **(A,B)** for MET values normalized to those of MOD **(C)** and for EMP values normalized to those of the MOD **(D)**. *p < 0.05, **p < 0.01, ***p < 0.001, ****p < 0.0001.

To verify the gene expression levels detected by the RNA-seq assay, we selected 17 DEGs (*Irs1, Akt2, Il6r, Jak1, Rptor*, and *Rheb* from the PI3K-Akt signaling pathway, *Ndufa2* and *Ndufa4* from oxidative phosphorylation, *Nsf* , *Cpped1, Atp2a2, Enpp2, Klf10*, and *Vav2* from metal ion binding, and *Pax5, Sox17* and *Sirt2* from negative regulation of transcription from the RNA polymerase II promoter) for qPCR (Figure 6). Four of those genes were upregulated, and six were downregulated by the MOD (Figures 6A,B). The qPCR results were consistent with the RNA-seq data. In contrast, the *Irs1, Il6r, Jak1, Ndufa2*, and *Ndufa4* genes were oppositely regulated by MET, and the *Nsf*, *Klf10, Sox17*, and *Pax5* genes were oppositely regulated by EMP, which were consistent with the RNA-seq results (Figures 6C,D). However, qPCR results showed that the *Cpped1, Atp2a2, Enpp2*, and *Vav2* genes were upregulated and that the *Sirt2* gene was downregulated by EMP, which was in contrast to the RNA-seq results.

## Discussion

As the major organ of carbohydrate and lipid biosynthesis in the body, the liver plays an important role in regulating glucose and lipid metabolism. Under normal circumstances, the liver can strictly control the balance of glucose and lipid metabolism through a variety of biological processes. However, in the case of T2D, hepatic carbohydrate and lipid biosynthesis fluxes increased, leading to hyperglycaemia and hypertriacylglycerolaemia (13). The current study has shown that when included in the standard treatment for T2D, EMP reduce liver fat in patients with non-alcoholic fatty liver disease (NAFLD) and T2D (14). In this study, we found that EMP treatment significantly reduces the fat vacuoles in the liver and decrease the levels of TG, CHO, NEFA, and LDL-c, and increase HDL-c level in serum when compared with the MOD group, indicating that EMP improves hepatic steatosis and ameliorate dyslipidemia in T2D. Furthermore, EMP treatment significantly reduced the levels of blood glucose and insulin in serum and improve glucose intolerance and insulin resistance in T2D rats, suggesting that EMP may also have a regulatory effect on hepatic glucose metabolism. In addition, compared with the MOD group, EMP treatment partially decrease the levels of TNF-α, IL-1β, IL-6, and IL-8 in serum. The above results indicate that, EMP treatment alleviates glucose intolerance, improves insulin resistance, ameliorates dyslipidaemia and has a slight effect on inflammation in T2D.

To further understand the mechanism by which EMP affects liver glucose metabolism, we used the RNA-seq method to analyze the liver transcriptome of Wistar rats and found 3,854 DEGs. Then, we performed GO and KEGG enrichment analysis on these DEGs. We found that MET treatment mainly affects the PI3K-Akt signaling pathway (Figure 4D) and Oxidative phosphorylation (Figure 5A), while the pathways affected by EMP are mainly concentrated in metal ion binding (Figure 4B) and negative regulation of transcription from the RNA polymerase II promoter (Figure 5C). The IRS1/PI3K/Akt signaling pathway is a vital pathway for regulating blood glucose balance through insulin and plays a key role in the development of T2D (15). Studies have shown that MET can upregulate the hepatic IRS2/PI3K/Akt signaling pathway, resulting in increased hepatic glycogen storage and improved insulin resistance (16). In this study, MET treatment upregulated the expression level of *Irs1* and downregulated most of the genes (such as *Il6r, Jak1*, and *Rptor*) in the PI3K-Akt signaling pathway, which may contribute to improvements in T2D.

GO analysis revealed that EMP treatment significantly downregulated genes in the metal ion binding pathway (Figure 4B), which involved in various biological functions, include lipid and glucose metabolism (such as *Klf10, Atp2a2*, and *Enpp2*), mitochondrial function (such as *Ndufs1, Oma1*, and *Pde12*) and β-cell function (such as *Atp2a2* and *Vav2*), et al. Our study showed that EMP treatment could significantly improve glucose tolerance and dyslipidemia, and alleviate insulin resistance in T2D rats (Table 2 and Figure 1C), which may be associated with the effect of EMP on the expression of related genes in the metal ion binding pathway. KLF10 is a member of the Krüppel-like family of transcription factors (Klfs), which has been shown to be implicated in many biological processes, including cell differentiation, apoptosis and glucose metabolism (17–20). In C57BL/6 mice, the mRNA and protein expression level of KLF10 was significantly increased in diet-induced non-alcoholic steatohepatitis and collagen producing activated hepatic stellate cells (21). *Klf10* expression was upregulated in the livers of diabetic and obese ob/ob mice and that knockdown of *Klf10* significantly inhibited the hepatic expression of gluconeogenic genes, decreased blood glucose levels and improved glucose tolerance (19). Our study showed that EMP treatment could obviously reduce the expression level of *Klf10* (Figures 4C, 6D). SERCA2b, one of the isoforms of SERCA2 that is encoded by the *Atp2a2* gene, plays an important role in regulating endoplasmic reticulum stress (ER stress), improving β-cell function and maintaining glucose homeostasis (22–24). Studies have shown that the protein and mRNA expression levels of SERCA2b are reduced in the liver of obese mice and that restoration of SERCA2b improves ER stress, increases glucose tolerance, and significantly reduces blood glucose levels; in addition, overexpression of SERCA2b in the liver of obese mice significantly reduces lipogenic gene expression and the triglyceride content in the liver (22). The present study showed that EMP treatment could affect the expression level of *Atp2a2*, and qPCR validation showed that EMP treatment could increase the expression level of *Atp2a2*. Furthermore, the upregulation of SERCA2 expression could increase insulin sensitivity and improve insulin resistance (25, 26), suggesting that the enhancement of insulin sensitivity of EMP may also related to the upregulated of *Atp2a2*.

VAV2 is a guanine nucleotide exchange factor for Ras-related C3 botulinum toxin substrate 1 (Rac1) that regulates glucose-stimulated insulin secretion (GSIS) in islet β-cell (27, 28). Our study demonstrated that EMP treatment significantly improved hyperinsulinemia (Figure 1D) and increased the expression level of *Vav2* in T2D rats (Figure 6D). These findings also suggest that EMP can affect GSIS through regulating the expression level of *Vav2*. Furthermore, recent studies also showed that VAV2 is associated with renal diseases and cardiovascular homeostasis (29, 30), which may be related to reduced cardiovascular risk in T2D. Moreover, *Cpped1* has been shown to be involved in adipocyte glucose metabolism, and knockdown of *Cpped1* expression in the human preadipocyte cell line SGBS increased the expression of genes involved in glucose metabolism and improved insulin-stimulated glucose uptake (31). Our results argued that EMP treatment significantly downregulated the expression level of *Cpped1*. ENPP2 is an important mediator of adipose tissue obesity, and adipocyte-specific deletion of *Enpp2* ameliorated glucose and insulin intolerance induced by an HFD (32). Besides, in elderly non-diabetic subjects with overweight or obesity, serum ATX (ENPP2 in rats) associated with the measures of glucose homeostasis and is an independent predictor of insulin sensitivity (33). Our present study argued that EMP treatment significantly ameliorates hepatic steatosis and insulin resistance (Figures 1C, 2). Therefore, our results suggested that EMP treatment could regulate the expression levels of genes in metal ion binding pathway that related to glucose and lipid metabolism (*Klf10, Atp2a2, Cpped1*, and *Enpp2*), β-cell function (*Atp2a2* and *Vav2*) and cardiovascular homeostasis (*Vav2*), which may contribute to regulate glucose and lipid metabolism and prevent cardiovascular risk in T2D rats.

Genes in the negative regulation of transcription from the RNA polymerase II promoter pathway (Figure 5C) are involved in glucose metabolism (*Sirt2*), adipogenesis (*Dact1*and *Gata2*), β-cell function (*Pik1* and *Jund*), and cardiovascular diseases (*Sox17* and *S100a1*), et al. Among those genes, *Sirt2* plays an important role in hepatic glucose uptake (34), and overexpression of *Sirt2* in hepatic could improve glucose tolerance and increase insulin sensitivity (34, 35). Our study showed that EMP treatment could affect the expression levels of *Sirt2* (Figures 5D, 6D). Dact1 is a preadipocyte gene that regulates adipogenesis through a coordinated effect on gene expression (36). Besides, Jund could regulate pancreatic β cell survival during metabolic stress (37). Our study showed that EMP treatment could significantly upregulate the expression levels of *Dac1* and *Jund* (Figure 5D). *Sox17*, one of the Sox (Sry-related HMG box) gene family members, has complex pleiotropic roles in regulating cardiovascular development (38, 39). Our study suggested that EMP obviously upregulated the expression level of *Sox17*. Furthermore, *Pax5* belongs to the family of paired-box domain transcription factors and is essential for B lymphocytes development (40, 41). In this study, EMP treatment significantly increased the expression level of *Pax5*. In short, current research suggests that EMP treatment could affect glucose metabolism and adipogenesis, pancreatic β cell function, and reduce cardiovascular risk by regulating gene expression in the negative regulation of transcription from the RNA polymerase II promoter pathway.

In conclusion, the present study demonstrates that EMP could increase insulin sensitivity, improve insulin resistance, alleviate hepatic steatosis and ameliorate glucose intolerance in T2D rats. Liver transcriptome analysis showed that EMP affects a large number of upregulated and downregulated genes. Some specific genes involved in metal ion binding pathway and the negative regulation of transcription from the RNA polymerase II promoter pathway are closely related to hepatic glucose and lipid metabolism. Our study provides new knowledge about the mechanism through which SGLT inhibitors can offer beneficial effects in type 2 diabetes and especially in the hepatic metabolism. A large number of genes found in this study also laid a solid foundation for further research on the new roles and/or mechanisms of EMP.

## Data Availability Statement

This article contains previously unpublished data. The data of RNA-seq can be found online at: https://www.ncbi.nlm.nih.gov/bioproject/PRJNA605030.

## Ethics Statement

The animal study was reviewed and approved by The animal experiments were performed in accordance with the Guidelines and Policies for Animal Surgery under the approval of the Chinese Academy of Medical Sciences and Peking Union Medical College, Beijing, China (approval No: SLXD-20170302526) and were approved by the Institutional Animal Use and Care Committee (IACUC).

## Author Contributions

BJ, SC, and PX designed the experiments. QL, LL, and JX performed the experiments. Data presentation and statistical analyses were performed by BJ, LL, and QL. QL wrote the manuscript.

### Conflict of Interest

The authors declare that the research was conducted in the absence of any commercial or financial relationships that could be construed as a potential conflict of interest.
