# Supplementary material for: Liver Transcriptomic Reveals Novel Pathways of Empagliflozin Associated With Type 2 Diabetic Rats
Source: Front Endocrinol (Lausanne). 2020 Mar 17;11:111. doi: 10.3389/fendo.2020.00111 (PMC7092631; doi:10.3389/fendo.2020.00111)
Supplement: Supplementary file 1 [file Image_1.pdf]

Supplementary Material

1. Supplementary Figures

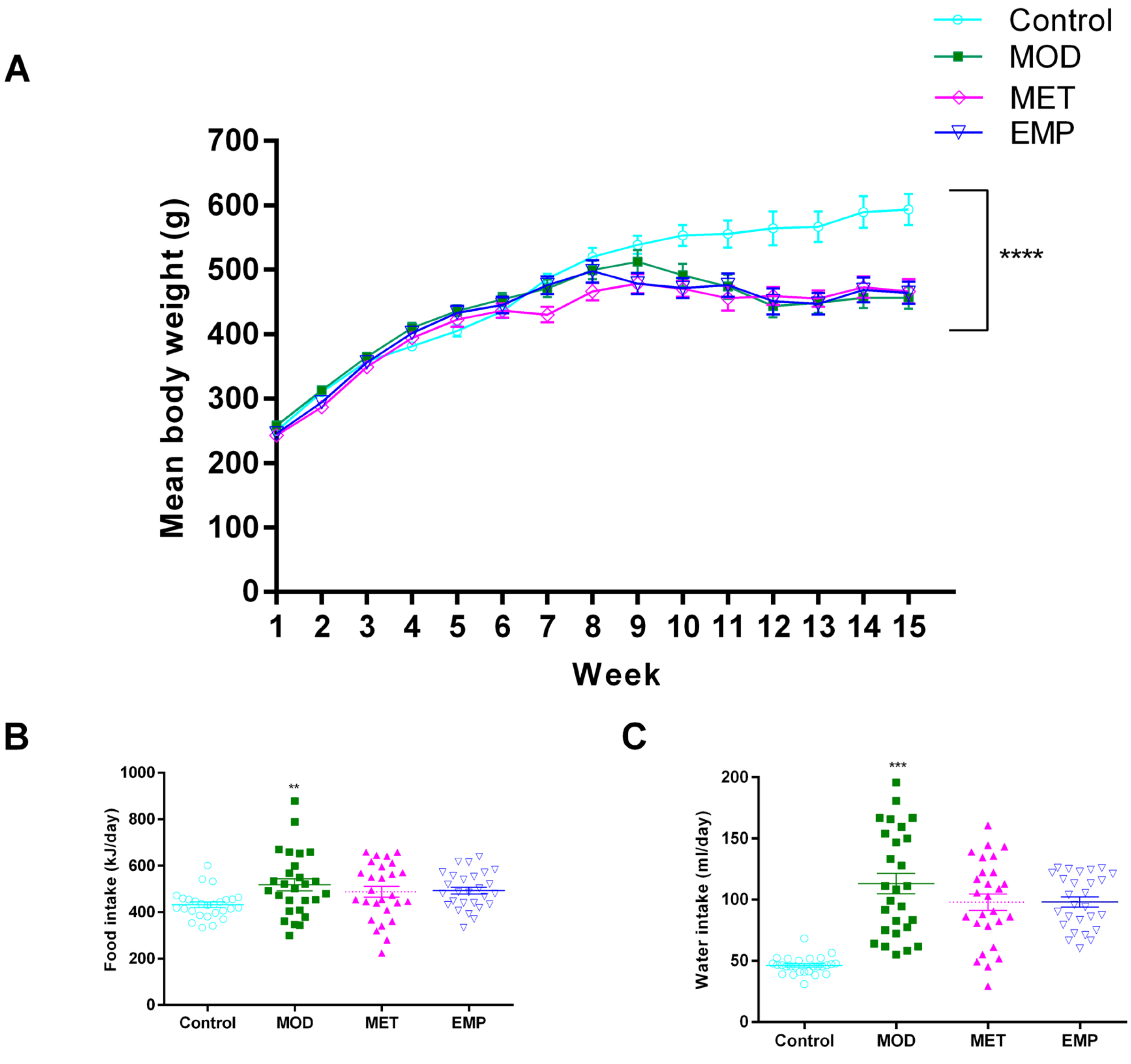

Supplementary Figure 1. The impact of MET and EMP on body weight, food intake and water intake. (A) Body wight. (B) Food intake. (C) Water intake. Notes: **p*<0.05, ***p*<0.01, ****p*<0.001, *****p*<0.0001 vs. Control. Data are presented as means ±SEM.
